# Supplementary material for: Diagnostic accuracy of non-invasive detection of SARS-CoV-2 infection by canine olfaction
Source: PLoS One. 2022 Jun 1;17(6):e0268382. doi: 10.1371/journal.pone.0268382 (PMC9159600; doi:10.1371/journal.pone.0268382)
Supplement: S1 Table — (DOCX) [file pone.0268382.s001.docx]

S1 Table: Diagnostic accuracy of canine detection as compared to the reference standard (nasopharyngeal NAAT, positivity defined as at least one target gene detected), according to medical history of COVID, having received treatment, smoking, the consumption of alcohol and coffee in the hours before testing.

| **Covariate** | **Total, n** | **Positive samples, n** | **Sensitivity**  **(95% CI*)** | **Specificity**  **(95% CI)** |
| --- | --- | --- | --- | --- |
| **Medical history of COVID** |  |  |  |  |
| Yes | 20 | 2 | 100% (16 to 100) | 83% (59 to 96) |
| No or unknown | 315 | 107 | 97% (92 to 99) | 92% (87 to 95) |
| **Having received medical treatment** |  |  |  |  |
| Yes | 120 | 46 | 100% (92 to 100) | 88% (78 to 94) |
| No | 215 | 63 | 95% (87 to 99) | 93% (87 to 96) |
| **Smocking in the last 24h** |  |  |  |  |
| Yes | 69 | 21 | 95% (76 to 100) | 94% (83 to 99) |
| No | 266 | 88 | 98% (92 to 100) | 90% (85 to 94) |
| **Alcohol in the last 24h** |  |  |  |  |
| Yes | 71 | 16 | 100% (79 to 100) | 93% (82 to 98) |
| No | 264 | 93 | 97% (91 to 99) | 91% (85 to 95) |
| **Coffee in the last hour** |  |  |  |  |
| Yes | 53 | 23 | 100% (85 to 100) | 90% (73 to 98) |
| No | 282 | 86 | 97% (86 to 99) | 91% (86 to 95) |
| **Gender** |  |  |  |  |
| Male | 165 | 60 | 95% (86 to 99) | 95% (89 to 98) |
| Female | 170 | 49 | 100% (93 to 100) | 88% (80 to 93) |

*95% CI : 95% Confidence Interval.
