# Supplementary material for: Diagnostic accuracy of non-invasive detection of SARS-CoV-2 infection by canine olfaction
Source: PLoS One. 2022 Jun 1;17(6):e0268382. doi: 10.1371/journal.pone.0268382 (PMC9159600; doi:10.1371/journal.pone.0268382)
Supplement: S2 Table — The others 2 dogs (Oska and Allo) tested only one PCR negative sweat sample each, both detected as negative. (DOCX) [file pone.0268382.s002.docx]

S2 Table: Diagnostic accuracy of the five individual dogs with sufficient samples as compared to the reference standard (nasopharyngeal NAAT, positivity defined as at least one target gene detected). The others 2 dogs (Oska and Allo) tested only one PCR negative sweat sample each, both detected as negative.

|  | **Total, n** | **Positive sample, n** | **Sensitivity**  **(95% CI*)** | **Specificity**  **(95% CI*)** |
| --- | --- | --- | --- | --- |
| **Dog 1 (Oxmo)** | 89 | 29 | 90% (73 to 98) | 95% (86 to 99) |
| **Dog 2 (Jinko)** | 203 | 73 | 100% (95 to 99) | 86% (79 to 92) |
| **Dog 3 (Leyko)** | 144 | 42 | 95% (84 to 99) | 91% (84 to 96) |
| **Dog 5 (Joye)** | 226 | 70 | 100% (95 to 100) | 90% (85 to 95) |
| **Dog E (Ortie)** | 23 | 7 | 71% (29 to 96) | 100% (79 to 100) |

*95% CI: 95% Confidence Interval
